# Complications of cricothyroidotomy versus tracheostomy in emergency surgical airway management: a systematic review

**DOI:** 10.1186/s12871-020-01135-2

**Published:** 2020-08-27

**Authors:** Fabricio Batistella Zasso, Kong Eric You-Ten, Michelle Ryu, Khrystyna Losyeva, Jaya Tanwani, Naveed Siddiqui

**Affiliations:** 1 MD, Department of Anaesthesia, Mount Sinai Hospital, University of Toronto, Toronto, Ontario Canada; 2 MLIS, Information Specialist, Sidney Liswood Health Science Library, Sinai Health System, University of Toronto, Toronto, Ontario Canada; 3 Summer Research Student, Mount Sinai Hospital, University of Toronto, Toronto, Ontario Canada; 4 Medical Student, Faculty of Medicine, University of Toronto, Toronto, Ontario Canada

**Keywords:** Cricothyroidotomy, Tracheostomy, Complications, Emergency surgical airway, Systematic review

## Abstract

**Background:**

Airway guidelines recommend an emergency surgical airway as a potential life-saving treatment in a “Can’t Intubate, Can’t Oxygenate” (CICO) situation. Surgical airways can be achieved either through a cricothyroidotomy or tracheostomy. The current literature has limited data regarding complications of cricothyroidotomy and tracheostomy in an emergency situation. The objective of this systematic review is to analyze complications following cricothyroidotomy and tracheostomy in airway emergencies.

**Methods:**

This synthesis of literature was exempt from ethics approval. Eight databases were searched from inception to October 2018, using a comprehensive search strategy. Studies were included if they were randomized controlled trials or observational studies reporting complications following emergency surgical airway. Complications were classified as minor (evolving to spontaneous remission or not requiring intervention or not persisting chronically), major (requiring intervention or persisting chronically), early (from the start of the procedure up to 7 days) and late (beyond 7 days of the procedure).

**Results:**

We retrieved 2659 references from our search criteria. Following the removal of duplicates, title and abstract review, 33 articles were selected for full-text reading. Twenty-one articles were finally included in the systematic review. We found no differences in minor, major or early complications between the two techniques. However, late complications were significantly more frequent in the tracheostomy group [OR (95% CI) 0.21 (0.20–0.22), *p* < 0.0001].

**Conclusions:**

Our results demonstrate that cricothyroidotomies performed in emergent situations resulted in fewer late complications than tracheostomies. This finding supports the recommendations from the latest Difficult Airway Society (DAS) guidelines regarding using cricothyroidotomy as the technique of choice for emergency surgical airway. However, emergency cricothyroidotomies should be converted to tracheostomies in a timely fashion as there is insufficient evidence to suggest that emergency cricothyrotomies are long term airways.

## Background

Airway management is an essential element of several medical specialties, including anesthesia, intensive care and emergency medicine. The vast majority of airways are managed uneventfully through basic and advanced use of available techniques and equipment. Failed airway management can lead to a “Can’t Intubate Can’t Oxygenate” (CICO) situation, which is defined by failed attempts to deliver oxygen to the patient by face-mask ventilation, tracheal intubation, and placement of a supraglottic airway [1–3]. CICO is a rare life-threatening situation which can result in significant morbidity and mortality leading to brain hypoxia or death, unless there is rapid resolution [4].

Airway management guidelines have been systematically developed to assist physicians in making decisions. An unanticipated difficult airway can lead to a CICO crisis. When this feared situation happens, airway guidelines recommend that an emergency surgical airway should be performed either through a cricothyroidotomy or tracheostomy [3, 5].

Historically, the guidelines progressed over the years on which technique should be used. In 1993, the first American Society of Anesthesiologists (ASA) guideline recommended that tracheostomy should be the surgical airway approach [6]. During the next decades, guidelines suggested that either cricothyroidotomy or tracheostomy could be performed [5, 7, 8]. The Difficult Airway Society (DAS) published their last guidelines recommending that cricothyroidotomy should be preferentially performed. This recommendation was founded on published evidence and where evidence is lacking, it was based on feedback from expert opinions. The guidelines were supported by the concept that a surgical airway should be a fast and simple procedure done with readily available equipment [3]. Moreover, an editorial by Pracy et al. advocated that anesthesiologists and head and neck surgeons should have a common surgical approach via the cricothyroid membrane [9]. This approach would increase the chances of success and decrease adverse patient outcomes. However, the DAS guidelines authors recognized that there is a lack of evidence in the literature on which one technique is superior to another [3].

Despite the recommendation towards cricothyroidotomy, the definitive technique for an emergency surgical airway is still debatable. The ideal approach should result in a high success rate and a low complication rate [10]. There are a considerable amount of studies analyzing the complications of elective and urgent surgical airways. However, the current literature has limited data regarding complications of cricothyroidotomy and tracheostomy in an emergency situation. Given the limited evidence supporting the preferred emergency surgical airway technique in the literature, we conducted a systematic review in order to compare the rate of complications in patients requiring an emergency cricothyroidotomy or tracheostomy. The goal of this systematic review was to compare the rates of early, late, minor and major complications between both techniques.

## Methods

This systematic review was exempt from ethics approval. The Preferred Reporting Items for Systematic Reviews and Meta-Analyses (PRISMA) guidelines were followed to conduct the systematic review of the literature [11].

### Study identification

The following electronic bibliographic databases were searched from inception to October 2018 using a comprehensive search strategy developed by an information specialist: (1) Ovid MEDLINE, (2) Ovid Embase, (3) Ovid EBM Reviews - Cochrane Central Register of Controlled Trials, (4) PubMed, and (5) EBSCO CINAHL Complete. We also searched the U.S. ClinicalTrials.gov, the World Health Organization’s International Clinical Trials Registry Platform (ICTRP), and the International Standard Randomised Controlled Trial Number Registry (ISRCTNR) for all registered clinical trials and randomized controlled trials (RCTs). A validated search filter for RCTs from the Cochrane Handbook for Systematic Reviews of Interventions [12] and observational studies of surgical interventions search filter by Fraser et al. [13] were used to screen Ovid Medline, Ovid Embase and PubMed. A pre-tested search filter for observational studies and RCTs was adapted from the Scottish Intercollegiate Guidelines Network [14] to screen EBSCO CINAHL Complete. Duplicate records were removed in EndNote X8 citation management software (search strategy detailed in Additional file 1).

The search strategy was structured according to the 2015 Peer-Reviewed Electronic Search Strategies (PRESS) Guidelines. We included Medical Subject Headings (MeSH), Entree terms, and free text terms related to ‘emergency medicine, ‘critical care’, ‘cricothyroidotomy’, and ‘tracheostomy’ and ‘postoperative complications’. No restrictions were applied to publication language or publication year. The search was complemented by hand-searching references of relevant articles, pre-register repositories (i.e. PROSPERO, Open Science Framework), and related organization websites.

### Eligibility criteria

Studies were considered eligible for inclusion if they were randomized controlled trials or observational studies reporting complications following emergency cricothyroidotomy or tracheostomy. Additionally, the studies were only included if they provided sufficient information to allow the reviewers to classify the type of complications. Studies were excluded if less than four patients were involved (considered case reports), complications for both techniques were reported together, complications for emergent and urgent procedures were not separated, or included patients below 10 years old. As this systematic review is focused on clinical practice, animal, cadaver, and mannequin studies were excluded.

### Data collection and data extraction

All article titles were screened. Abstracts of potentially relevant articles were subsequently assessed, and those without relevance were eliminated. Full-text manuscripts of all remaining studies were obtained, read and assessed qualitatively. Disagreements between the authors were resolved by a consensus-based discussion. The risk of bias was assessed using the modified Newcastle-Ottawa Scale (NOS). This scale evaluates the quality of studies through three items (selection, comparability and outcome), resulting in a grade between one and eight. We considered articles with less than four points as high-risk for bias, which was the criteria for exclusion. NOS scores of selection, comparability and outcome for each study are included in Table 1.

**Table 1:** Newcastle-Ottawa Scale Scores (Scores between 1 and 8)

Source		Selection			Comparability		Outcome		Total
	Representative-ness of the intervention cohort	Selection of non-intervention cohort	Ascertainment of intervention	Outcome of interest not present at start of study	Cohort comparable on the basis of the design	Assessment of outcome	Follow-up long enough	Adequacy of follow-up	
Waldron et al., 1990 [15]	No	No	Yes	Yes	No	Yes	Yes	Yes	5
Ben-Nun et al., 2004 [16]	Yes	No	Yes	Yes	No	Yes	Yes	Yes	6
Davidson et al., 2012 [17]	Yes	No	Yes	Yes	No	Yes	No	No	4
Muhammad et al., 2012 [18]	Yes	No	Yes	Yes	No	Yes	No	Yes	5
Fang et al., 2015 [19]	Yes	No	Yes	Yes	No	Yes	No	No	4
Miklus et al., 1989 [20]	Yes	No	Yes	Yes	No	Yes	No	No	4
Cook et al., 1991 [21]	Yes	No	Yes	Yes	No	Yes	Yes	Yes	6
Nugent et al., 1991 [22]	Yes	No	Yes	Yes	No	Yes	No	No	4
Boyle et al., 1993 [23]	Yes	No	Yes	Yes	No	Yes	No	Yes	5
Hawkins et al., 1995 [24]	Yes	No	Yes	Yes	No	Yes	Yes	No	5
Jacobson et al., 1996 [25]	Yes	No	Yes	Yes	No	Yes	Yes	No	5
Isaacs et al., 1997 [26]	Yes	No	Yes	Yes	No	Yes	Yes	No	5
Leibovici et al., 1997 [27]	Yes	No	Yes	Yes	No	Yes	No	No	4
Wright et al., 2003 [28]	Yes	No	Yes	Yes	No	Yes	Yes	No	5
Bair et al., 2003 [29]	Yes	No	Yes	Yes	No	Yes	No	Yes	5
McIntosh et al., 2008 [30]	Yes	No	Yes	Yes	No	Yes	No	Yes	5
Warner et al., 2009 [31]	Yes	No	Yes	Yes	No	Yes	No	Yes	5
King et al., 2012 [32]	Yes	No	Yes	Yes	No	Yes	No	No	4
Darby et al., 2016 [33]	Yes	No	Yes	Yes	No	Yes	No	No	4
Gillespie et al., 1999 [34]	Yes	Yes	Yes	Yes	No	Yes	Yes	No	6
Beshey et al., 2014 [35]	Yes	Yes	Yes	Yes	Yes	Yes	No	No	6

Each category is graded with a score of 1 (Yes) or 0 (No)

Complications were classified as minor (evolving to spontaneous remission or not requiring intervention or not persisting chronically), major (requiring intervention or persisting chronically), early (from the start of the procedure up to 7 days) and late (beyond 7 days of the procedure). A full description of minor and major complications is described in Additional file 2. The list of complications was compiled pre-screening of the articles.

We extracted the following data from the included studies: type of study, follow-up period, sample size, number of minor complications, number of major complications, number of early complications, sample size for late complications and number of late complications. We gathered each type of complications in two groups: CRICO (cricothyroidotomy) and TRACH (tracheostomy). A number of articles did not performed follow-up, which resulted in no late complications reported. In the articles that performed it, there were lost due to death or failure to contact the patients. As a result, the sample size for late complications was smaller than the sample size for early complications.

### Statistical analysis

We compared minor, major, early and late complications between cricothyroidotomy and tracheostomy groups using the chi-square test. Additionally, we reported the pooled risk ratio with 95% confidence intervals using weighted logistic regression to compare the two techniques (the weight was defined as the ratio of the sample size for each study and the total sample size of all studies). A difference of *p* < 0.05 was considered statistically significant in all analyses.

## Results

**Fig. 1:**
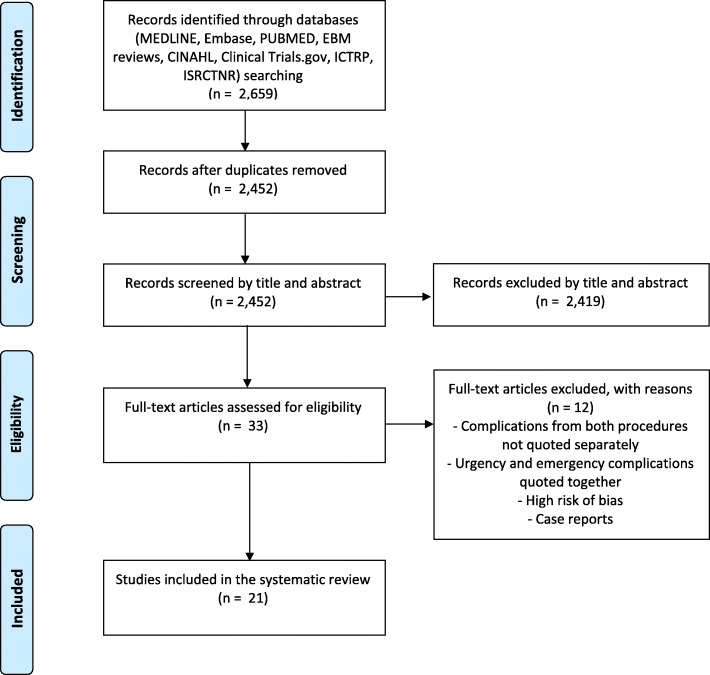
PRISMA flow chart of the study. Abbreviations: ICTRP = World Health Organization’s International Clinical Trials Registry Platform, ISRCTNR = International Standard Randomised Controlled Trial Number Registry. To be positioned in the Results section

We retrieved 2659 references from our databases search. After removing duplicates, 2452 records were obtained. Following the title and abstract review, 33 articles were selected for full-text reading. Twelve articles were excluded as they did not meet the study criteria. Therefore, 21 articles were included in the systematic review (Fig. 1 – Flow chart).

We found 20 observational studies (19 retrospective and one prospective), and one randomized clinical trial (RCT) describing complications following emergency surgical airway. The number of studies in each category is not additive. Five studies reported only tracheostomy complications [15–19]; 14 studies reported only cricothyroidotomy complications [20–33]; and two studies reported complications from both techniques (RCT included) [34, 35].

Two articles from the TRACH group included elective, urgent and emergent procedures [15, 18], but only data from the latter was extracted (Table 2). The remaining articles contained only emergency procedures. In the TRACH group, three articles performed percutaneous [16, 17, 35], and four performed surgical approach [15, 18, 19, 34]. In the CRICO group, only one article used percutaneous [35], one used both (13 procedures under percutaneous and 16 under surgical) [27], and all other used surgical technique [20–26, 28–34] (Table 3).

**Table 2:** Studies reporting complications after emergency Tracheostomy (TRACH group)

Author, Yr	Type of study	Type of procedure	Type of technique	Setting; Performers	Follow-up period	Sample size	Complications				Sample size for Late complications	Description of Late complications
							Minor	Major	Early	Late		
Waldron et al., 1990 [15]	Retrospective	Elective, urgency, emergency	Surgical	OR; ENT	Minimum 6 months	38	5	4	7	2	38	Tracheo-cutaneous fistula, scar formation
Gillespie et al., 1999 [34]	Retrospective	Emergency	Surgical	Intra-hospital; ENT, general surgeon	Average 23 months	14	1	2	3	0	8	–
Ben-Nun et al., 2004 [16]	Retrospective	Emergency	Percutaneous	ED, ICU; Thoracic surgeons	1 year	10	1	0	0	1	5	Recurrent hemoptysis
Davidson et al., 2012 [17]	Retrospective	Emergency	Percutaneous	Intra-hospital; Trauma surgeons	NA	18	0	1	1	NA	NA	–
Muhammad et al., 2012 [18]	Prospective cohort	Elective, urgency, emergency	Surgical	OR; ENT	7 days	50	17	11	28	NA	NA	–
Beshey et al., 2014 [35]	RCT	Emergency	Percutaneous	ED, ICU; Physician	Maximum 48 h	84	1	2	3	NA	NA	–
Fang et al., 2015 [19]	Retrospective	Emergency	Surgical	OR, bedside; ENT	Mean 7.2 weeks	68	10	29	11	28	49	Bleeding (5), tracheitis (5), obstructed tube (4), tube dislodgement (4), pneumonia (3), neck abscess (3), pneumonia (2), tracheal granuloma (2)
**TOTAL**						**282**	**35**	**49**	**53**	**31**	**100**	

*OR* Operation Room, *ENT* Ear, Nose, Throat, *ED* Emergency Department, *NA* Data Not Available, *ICU* Intensive Care Unit, *RCT* Randomized Clinical Trial

**Table 3:** Studies reporting complications after emergency Cricothyroidotomy (CRICO group)

Author, Yr	Type of study	Type of procedure	Type of technique	Setting; Performers	Follow-up period	Sample size	Complication				Sample size for Late complications	Description of Late complications
							Minor	Major	Early	Late		
Miklus et al., 1989 [20]	Retrospective	Emergency	Surgical	Pre-hospital; Physicians	NA	20	0	0	0	0	8	–
Cook et al., 1991 [21]	Retrospective	Emergency	Surgical	Pre-hospital; Nurse/Paramedics	24 h	68	0	3	3	NA	NA	–
Nugent et al., 1991 [22]	Retrospective	Emergency	Surgical	Pre-hospital; Nurses	NA	55	0	11	9	2	15	Subglottic stenosis (2)
Boyle et al., 1993 [23]	Retrospective	Emergency	Surgical	Pre-hospital; Nurses	NA	69	0	6	6	NA	NA	
Hawkins et al., 1995 [24]	Retrospective	Emergency	Surgical	Pre-hospital; Not given (*n* = 8) ED; Trauma surgeons (*n* = 58)	5 years	66	0	3	3	0	26	–
Jacobson et al., 1996 [25]	Retrospective	Emergency	Surgical	Pre-hospital; Paramedics	2 to 5 years	50	5	6	11	0	19	–
Isaacs et al., 1997 [26]	Retrospective	Emergency	Surgical	Intra-hospital; ENT	Average 8 months	65	0	13	10	3	27	Cartilage injury, tracheal granuloma, vocal cord paralysis
Leibovici et al., 1997 [27]	Retrospective	Emergency	Percutaneous (*n* = 13), surgical (*n* = 16)	Pre-hospital; Physicians	NA	29	3	4	7	0	13	–
Gillespie et al., 1999 [34]	Retrospective	Emergency	Surgical	Intra-hospital; ENT, general surgeon	Average 23 months	20	2	2	3	1	12	Subglottic stenosis
Wright et al., 2003 [28]	Retrospective	Emergency	Surgical	ED; Surgeons	Minimum 6 months	46	0	7	2	5	15	Pneumonia (4), retropharyngeal abscess
Bair et al., 2003 [29]	Retrospective	Emergency	Surgical	Pre-hospital; Nurses (*n* = 22) ED; EM, Trauma surgeon (*n* = 28)	NA	50	40	15	55	NA	NA	–
McIntosh et al., 2008 [30]	Retrospective	Emergency	Surgical	Pre-hospital; Nurse/Paramedics	NA	17	0	5	5	NA	NA	–
Warner et al., 2009 [31]	Prospective Cohort	Emergency	Surgical	Pre-hospital; Paramedics	NA	11	0	3	3	NA	NA	–
King et al., 2012 [32]	Retrospective	Emergency	Surgical	Pre-hospital; Nurse / Paramedics (*n* = 6) ED; Surgeons (*n* = 48)	NA	54	2	9	11	NA	NA	–
Beshey et al., 2014 [35]	RCT	Emergency	Percutaneous	ED, ICU; Physician	Maximum 48 h	85	2	12	14	NA	NA	–
Darby et al., 2016 [33]	Retrospective	Emergency	Surgical	Intra-hospital; ICU physicians	NA	20	11	20	31	NA	NA	–
**TOTAL**						**725**	**65**	**119**	**173**	**11**	**135**	

*NA* Data Not Available, *ED* Emergency Department, *ENT* Ear, Nose, Throat, *EM* Emergency Medicine physician, *RCT* Randomized Clinical Trial, *ICU* Intensive Care Unit

The location and healthcare providers performing the emergency surgical airways varied between tracheostomies and cricothyroidotomies. All emergency tracheostomies were performed in the hospital, in settings such as Operation Room (OR), Emergency Department (ED), Intensive Care Unit (ICU), and on Inpatient Unit, mainly by surgeons (ENT - Ear, Nose, Throat, thoracic, general, trauma) [15–19, 34, 35]. In contrast, cricothyroidotomies were performed in both pre- and intra-hospital by a variety of healthcare workers including physicians and non-physicians. 50% of the 16 included studies reported only pre-hospital cricothyroidotomies. In six out of eight pre-hospital studies, non-physicians (nurses and/or paramedics) performed the surgical procedures [21–23, 25, 30, 31], whereas physicians executed the pre-hospital interventions in the remaining two studies [20, 27]. Five studies reported only intra-hospital emergency cricothyroidotomies performed by different physicians (ENT, general surgeons, ICU physicians) and in different locations (ER, OR, Inpatient Unit). Three studies described a mix of pre- and intra-hospital cricothyroidotomies [24, 29, 32]; intra-hospital cases were performed in the ED by trauma surgeons or emergency medicine (EM) physicians whereas nurses or paramedics carried out pre-hospital procedures. The third study did not specify location of emergency cricothyroidotomies.

There was a marked heterogeneity in the follow-up periods. In the TRACH group, they ranged from a maximum of 48 h to 23 months, and one article did not report for how long patients were followed [17]. In the CRICO group, the follow-up period varied from 24 h to 5 years. However, nine articles did not describe it [20, 22, 23, 27, 29–33]. Two articles still reported late complications despite not specifying for how long patients were followed [22, 27].

In the TRACH group, the number of procedures per study ranged from 10 to 84, with a total sample size of 282, whereas the number of interventions per study varied from 11 to 85, with a total a sample size of 725 in the CRICO group. There were no statistical differences in minor, major and early complications between TRACH and CRICO groups (Table 3). However, the CRICO group showed fewer late complications compared to TRACH group [OR (95% CI) 0.21 (0.20–0.22), *p* < 0.0001] (Table 4).

**Table 4:** Comparison between Cricothyroidotomy (CRICO group) and Tracheostomy (TRACH) complications

Complications	CRICO Number of complications/ total sample size (%)	TRACH Number of complications/ total sample size (%)	*p*-value 1	CRICO vs TRACH OR (95%CI)	*p*-value 2
Minor^a^	65/725 (8.97%)	35/282 (12.41%)	0.1	0.60 (0.08, 4.25)	0.61
Major^b^	119/725 (16.41%)	49/282 (17.38%)	0.71	0.77 (0.16, 3.64)	0.74
Early^c^	173/725 (23.86%)	53/282 (18.79%)	0.08	0.72 (0.14, 3.63)	0.69
Late^d^	11/135 (8.15%)	31/100 (31.00%)	< 0.0001	0.21 (0.20, 0.22)	< 0.0001

^a^Complications evolving to spontaneous remission or not requiring intervention or not persisting chronically

^b^Complications requiring intervention or persisting chronically

^c^Complications from the start of the procedure up to 7 days

^d^Complications beyond 7 days of the procedure

A full description of minor and major complications is described in Additional file 2

*Abbreviations*: *OR* Odds Ratio, *CI* Confidence Interval

The reported *p*-value 1 was based on the comparisons of outcomes between two groups using Chi-squares where the number of events and total sample size were obtained by pooling all the studies

The reported *p*-value 2 was based on the weighted logistic regression, where the weight was defined based on the sample size of each study

## Discussion

Our systematic review summarized 21 articles from the literature that described complications following surgical airways performed under emergency situations. Our results demonstrated that there was a higher rate of late complications in tracheostomies than in cricothyroidotomies performed in the emergency setting. When comparing minor, major and early complications, there were no differences between the two procedures.

To our knowledge, this is the first systematic review comparing complications of cricothyroidotomies and tracheostomies in emergencies. The majority of the studies assessing complications of both techniques was either elective or urgent procedures. A previous prospective study comparing elective surgical tracheostomy and cricothyroidotomy in the ICU showed that the incidence of minor, major, early, and late complications was similar between both techniques [36]. One possible explanation for the different findings was the emergent nature of the cases in our review. Factors related to emergency procedures, such as time constraints, higher complexity, and higher tissue trauma, can lead to late complications.

The description of complications and safety of cricothyroidotomies were based in non-emergency cases for decades. The cricothyroidotomy technique was introduced a long time after the first tracheostomy was performed. In 1909, Chevelier Jackson described the surgical approach and considerations to perform cricothyroidotomies successfully. In 1921, he published a case series of 200 patients with tracheal stenosis, showing that 158 had undergone cricothyroidotomies. He concluded that this technique had a high risk of complications compared to tracheostomies [37]. In 1976, Brantigan and Grow published a case series of 655 patients that undergone elective cricothyroidotomies. The overall complication rate was 6.1%, and only eight patients developed subglottic stenosis [38]. Thereafter, several subsequent case series reporting low rates of post-procedure complications again popularized the use of cricothyroidotomy as a method of surgical airway management. Our findings aligned with this trend, reassuring the safety of this technique for emergency surgical airway.

CICO is a rare emergency and there is limited evidence of its incidence. Based on a few cohort studies and the National Audit Project 4 of severe airway emergencies, CICO incidence is around 1:10,000 to 1:50,000 of general anesthetics. Furthermore, there is additional evidence that it may be up to 10 times more frequent in settings outside of the operating room such as intensive care and the emergency department [1, 2, 39].. Despite emergency cricothyroidotomy being a relatively simple procedure, it is quite rare, with rates ranging from 0.2 to 1.2%, considering all tracheal intubations [40–43]. None of the retrieved articles in this review for either group had the procedures performed by anesthesiologists. This was expected for the TRACH group but was surprising for the CRICO group, as most anesthesiologists have either limited or no experience in performing a tracheostomy [9]. Possible explanations are that recommendation for anesthesiologists to perform cricothyroidotomy as Front Of Neck Access (FONA) is recent or anesthesiologists are only publishing case reports as they rarely perform it during their careers or that the anesthesiologists are still not comfortable performing this procedure [4, 44]. A recent editorial stated that there are many CICO situations in which trained surgeons are not readily available, and that the anesthesiologists, who are airway experts, should perform the emergency cricothyroidotomy [45]. Hence it is imperative that anesthesiologists be competent in this technique. To achieve an adequate level of comfort and competence, hands-on training in cricothyrotomy is essential [46].

There are several limitations to our systematic review that deserve consideration. The studied population is particularly sensitive to loss of follow-up, which reduces the number of patients available for assessment and analysis of late complications. A review of medical-legal claims from the United States highlighted that most FONAs were carried out peri-cardiac arrest or death [47]. Therefore, the number of patients who die immediately or in the first weeks after the event is high. Another limitation is that we did not compare outcome measures with the operators and the context of the situations. However, many factors of an operator including experience, age, speciality training, technical and nontechnical skills and factors of context including poor lighting, support and space will be difficult to analyze properly without unbiased evidence. A third limitation is that some papers that were included in our review had poorly defined complications. However, we have decided to include these publications since the complications were also defined as short and long term, consistent with the other papers. Hence, it would be very challenging to base a conclusion of the review on well-defined complications, which were highly variable amongst different authors.

This systematic review was mainly based on observational studies. For ethical and practical reasons, we expected few, if any, randomized controlled trials to have been conducted. This expectation was proven correct, as we found only one RCT [35]. We found a common lack of standardization. There was a wide range in the length of follow-up. Several articles only reported early complications. Others described late complications but did not define the follow-up period. Additionally, we observed that some articles had a higher incidence of complications than others. A possible reason is a difference in the methodology for their identification. A strength of this retrospective review is that it can accumulate data for a large number of patients for a rare event. However, a limitation of our study is the possibility of selection biases, caused by high heterogeneity between the studies analyzed. This issue has been quoted in previous reviews about emergency cricothyroidotomy [48–50]. Future studies should focus on clearly defined and described criteria to determine complications after an emergent surgical airway placement.

## Conclusion

In conclusion, this systematic review demonstrates that emergency tracheostomies are associated with a greater incidence of late complications than emergency cricothyrotomies. These findings support the recommendations from the latest DAS guidelines that cricothyroidotomy performed by anesthesiologists is the technique of choice for emergency surgical airway. However, there is insufficient evidence to suggest that emergency cricothyrotomies are long term airways. Current practices remain that emergency cricothyrotomies are short term airways that should be converted to tracheostomies in a timely fashion. Future studies in emergency cricothyrotomies as long term airways are warranted.

## Supplementary information

**Additional file 1.**

**Additional file 2.**

## Data Availability

The datasets used and/or analysed during the current study are available from the corresponding author on reasonable request.

## Abbreviations

ASA: American Society of Anesthesiologists
CICO: Can’t Intubate, Can’t Oxygenate
DAS: Difficult Airway Society
CRICO: Cricothyroidotomy
ED: Emergency department
EM: Emergency medicine
ENT: Ear, Nose, Throat
FONA: Front Of Neck Access
ICU: Intensive care unit
MeSH: Medical Subject Headings
NOS: Newcastle-Ottawa Scale
OR: Operation room
PRISMA: The Preferred Reporting Items for Systematic Reviews and Meta-Analyses
PRESS: Peer-Reviewed Electronic Search Strategies
RCT: Randomized clinical trial
TRACH: Tracheostomy
