# Supplementary material for: Complications of cricothyroidotomy versus tracheostomy in emergency surgical airway management: a systematic review
Source: BMC Anesthesiol. 2020 Aug 27;20:216. doi: 10.1186/s12871-020-01135-2 (PMC7450579; doi:10.1186/s12871-020-01135-2)
Supplement: Supplementary file 1 — Additional file 1. [file 12871_2020_1135_MOESM1_ESM.docx]

**Complications of cricothyroidotomy versus tracheostomy in emergency surgical airway management: A systematic review**

**1. Databases/Sources Screened**

1. 1) MEDLINE
2. 2) Embase
3. 3) EBM Reviews – Cochrane Centralized Registered Trials
4. 4) EBM Reviews – Cochrane Database of Systematic Reviews
5. 5) PubMed
6. 6) CINAHL
7. 7) ClinicalTrials.gov
8. 8) ICTRP
9. 9) ISRCTNR

**searched August 28 - September 27, 2018*

**2. Search Strategy**

Database: All Ovid MEDLINE(R) <1946 to Present> Search Strategy:

--------------------------------------------------------------------------------

1 Emergency Medicine/ (12399)

2 exp Emergency Nursing/ (6785)

3 exp Emergencies/ (38543)

4 Emergency Medical Services/ (39436)

5 exp Emergency Service, Hospital/ (68188)

6 Emergency Treatment/ (10079)

7 Critical Care/ (47797)

8 exp Critical Care Nursing/ (1490)

9 Ambulatory Care/ (40651)

10 Ambulatory Care Facilities/ (17245)

11 Intensive Care Units/ (47985)

12 exp Respiratory Care Units/ (582)

13 (emergen* or urgent care or critical* or intensive* or trauma* or ambulatory or ICU* or CCU* or immediate*).tw,kf,kw. (1899216)

14 (cricothyroidotom* or crico-thyroidotom*).tw,kf,kw. (456)

15 (thyrocricotom* or thyro-cricotom*).tw,kf,kw. (0)

16 (cricothyrotom* or crico-thyrotom*).tw,kf,kw. (369)

17 coniotom*.tw,kf,kw. (53)

18 ((cricothyroid or crico-thyroid or subthyroid or sub-thyroid) adj2 (incis* or cut* or punctur* or dissect*)).tw,kf,kw. (48)

19 exp Tracheostomy/ (6766)

20 exp Tracheotomy/ (8218)

21 (tracheotom* or tracheostom*).tw,kf,kw. (18741)

22 (minitracheostom* or mini-tracheostom*).tw,kf,kw. (83)

23 (minitracheotom* or mini-tracheotom*).tw,kf,kw. (79)

24 or/1-13 (2005530)

25 or/14-23 (24549)

26 Comparative studies/ (1812080)

27 Follow-up studies/ (600555)

28 Time factors/ (1134738)

29 (preoperat$ or pre operat$).mp. (310588)

30 Chang$.tw. (2764501)

31 Evaluat$.tw. (3025483)

32 Reviewed.tw. (455588)

33 Prospective$.tw. (606255)

34 Retrospective$.tw. (606869)

35 Baseline.tw. (477181)

36 Cohort.tw. (422731)

37 Case series.tw. (60239)

38 or/26-37 (8648953)

39 randomized controlled trial.pt. (470110)

40 controlled clinical trial.pt. (92712)

41 randomized.ab. (414854)

42 placebo.ab. (189645)

43 drug therapy.fs. (2056035)

44 randomly.ab. (293666)

45 trial.ab. (432415)

46 groups.ab. (1808286)

47 or/39-46 (4252598)

48 exp animals/ not humans.sh. (4507542)

49 47 not 48 (3669356)

50 38 or 49 (10448025)

51 24 and 25 and 50 (4131)

52 limit 51 to "all adult (19 plus years)" (2388)

53 remove duplicates from 52 (2388)

54 Postoperative Complications/ (338341)

55 (complication* or (adverse adj2 (effect* or event* or condition*))).tw,kw,kf. (1141047)

56 54 or 55 (1355774)

57 53 and 56 (1102)

***************************

Database: Embase Classic+Embase <1947 to 2018 October 24> Search Strategy:

--------------------------------------------------------------------------------

1 emergency medicine/ (37676)

2 emergency treatment/ or emergency care/ (55220)

3 emergency health service/ or hospital emergency service/ (91960)

4 emergency nursing/ (6214)

5 emergency/ (57076)

6 intensive care/ or intensive care nursing/ (118744)

7 intensive care unit/ or medical intensive care unit/ or surgical intensive care unit/ (136317)

8 ambulatory care/ or ambulatory care nursing/ (36244)

9 outpatient care/ or outpatient department/ (107359)

10 (emergen* or urgent care or critical* or intensive* or trauma* or ambulatory or ICU* or CCU* or immediate*).tw,kw. (2644283)

11 (cricothyroidotom* or crico-thyroidotom*).tw,kw. (690)

12 (thyrocricotom* or thyro-cricotom*).tw,kw. (1)

13 (cricothyrotom* or crico-thyrotom*).tw,kw. (570)

14 coniotom*.tw,kw. (70)

15 ((cricothyroid or crico-thyroid or subthyroid or sub-thyroid) adj2 (incis* or cut* or punctur* or dissect*)).tw,kw. (66)

16 exp Tracheostomy/ (22783)

17 exp Tracheotomy/ (14780)

18 (tracheotom* or tracheostom*).tw,kw. (29554)

19 (minitracheostom* or mini-tracheostom*).tw,kw. (96)

20 (minitracheotom* or mini-tracheotom*).tw,kw. (93)

21 or/1-10 (2865762)

22 or/11-20 (43274)

23 crossover-procedure/ (57298)

24 double-blind procedure/ (156688)

25 randomized controlled trial/ (520844)

26 single-blind procedure/ (32776)

27 (random* or factorial* or crossover* or cross over* or placebo* or (doubl* adj blind*) or (singl* adj blind*) or assign* or allocat* or volunteer*).tw. (1994257)

28 or/23-27 (2090038)

29 exp cohort analysis/ (409376)

30 exp longitudinal study/ (118253)

31 exp prospective study/ (479484)

32 exp follow up/ (1362346)

33 cohort$.tw. (800119)

34 exp case control study/ (149566)

35 (case$ and control$).tw. (670402)

36 exp case study/ (66233)

37 (case$ and series).tw. (265706)

38 case report/ (2385872)

39 (case$ adj2 report$).tw. (732584)

40 (case$ adj2 stud$).tw. (289324)

41 or/29-40 (5535601)

42 28 or 41 (7186535)

43 21 and 22 and 42 (7465)

44 limit 43 to (adult <18 to 64 years> or aged <65+ years>) (4312)

45 remove duplicates from 44 (4268)

46 complication/ (139723)

47 adverse event/ (19147)

48 (complication* or (adverse adj2 (effect* or event* or condition*))).tw,kw. (1699405)

49 46 or 47 or 48 (1755309)

50 45 and 49 (1433)

***************************

Database: EBM Reviews - Cochrane Central Register of Controlled Trials <September 2018> Search Strategy:

--------------------------------------------------------------------------------

1 exp Emergency Medicine/ (234)

2 exp critical care nursing/ or exp emergency nursing/ (95)

3 exp emergencies/ (1044)

4 emergency medical services/ or exp advanced trauma life support care/ or exp emergency service, hospital/ (2883)

5 Emergency Treatment/ (235)

6 critical care/ or intensive care/ (1569)

7 intensive care units/ or exp respiratory care units/ (1991)

8 ambulatory care/ (3032)

9 Ambulatory Care Facilities/ (425)

10 (emergen* or urgent care or critical* or intensive* or trauma* or ambulatory or ICU* or CCU* or immediate*).tw,kw. (133949)

11 (cricothyroidotom* or crico-thyroidotom*).tw,kw. (35)

12 (thyrocricotom* or thyro-cricotom*).tw,kw. (0)

13 (cricothyrotom* or crico-thyrotom*).tw,kw. (39)

14 coniotom*.tw,kw. (1)

15 ((cricothyroid or crico-thyroid or subthyroid or sub-thyroid) adj2 (incis* or cut* or punctur* or dissect*)).tw,kw. (6)

16 exp Tracheostomy/ (157)

17 exp Tracheotomy/ (69)

18 (tracheotom* or tracheostom*).tw,kw. (782)

19 (minitracheostom* or mini-tracheostom*).tw,kw. (4)

20 (minitracheotom* or mini-tracheotom*).tw,kw. (6)

21 or/1-10 (137159)

22 or/11-20 (858)

23 21 and 22 (516)

24 (infant* or neonat* or newborn* or p?ediatric* or child*).tw,kw. (128802)

25 23 not 24 (482)

26 remove duplicates from 25 (453)

27 postoperative complications/ (15694)

28 (complication* or (adverse adj2 (effect* or event* or condition*))).tw,kw. (158308)

29 27 or 28 (167538)

30 26 and 29 (190)

31 remove duplicates from 30 (190)

32 from 30 keep 1-190 (190)

***************************

Database: EBM Reviews - Cochrane Database of Systematic Reviews <2005 to October 24, 2018> Search Strategy:

--------------------------------------------------------------------------------

1 (emergen* or urgent care or critical* or intensive* or trauma* or ambulatory or ICU* or CCU* or immediate*).tw,kw. (6960)

2 (cricothyroidotom* or crico-thyroidotom*).tw,kw. (1)

3 (thyrocricotom* or thyro-cricotom*).tw,kw. (0)

4 (cricothyrotom* or crico-thyrotom*).tw,kw. (1)

5 coniotom*.tw,kw. (0)

6 ((cricothyroid or crico-thyroid or subthyroid or sub-thyroid) adj2 (incis* or cut* or punctur* or dissect*)).tw,kw. (0)

7 (tracheotom* or tracheostom*).tw,kw. (76)

8 (minitracheostom* or mini-tracheostom*).tw,kw. (3)

9 (minitracheotom* or mini-tracheotom*).tw,kw. (0)

10 or/2-9 (78)

11 1 and 10 (66)

12 (infant* or neonat* or newborn* or p?ediatric* or child*).tw,kw. (5523)

13 11 not 12 (25)

14 remove duplicates from 13 (25)

15 (complication* or (adverse adj2 (effect* or event* or condition*))).tw,kw. (8619)

16 14 and 15 (23)

***************************

**PubMed**

**((((("postoperative complications"[MeSH Terms]) OR ((complication*[Title/Abstract] OR "adverse effect"[Title/Abstract] OR "adverse effects"[Title/Abstract] OR "adverse event"[Title/Abstract] OR "adverse events"[Title/Abstract] OR "adverse condition"[Title/Abstract] OR "adverse conditions"[Title/Abstract])))) AND (((((((((((((((((("emergency medicine"[MeSH Terms]) OR "emergency nursing"[MeSH Terms]) OR emergencies[MeSH Terms]) OR emergency medical service[MeSH Terms]) OR emergency service, hospital[MeSH Terms]) OR emergency treatment[MeSH Terms]) OR "critical care"[MeSH Terms]) OR critical care nursing[MeSH Terms]) OR "ambulatory care"[MeSH Terms]) OR ambulatory care facilities[MeSH Terms]) OR intensive care unit[MeSH Terms]) OR "respiratory care units"[MeSH Terms]) OR ((emergen*[Title/Abstract] OR "urgent care"[Title/Abstract] OR critical*[Title/Abstract] OR intensive*[Title/Abstract] OR trauma*[Title/Abstract] OR ambulatory[Title/Abstract] OR ICU*[Title/Abstract] OR CCU*[Title/Abstract] OR immediate*[Title/Abstract])))) AND ((((((((((((cricothyroidotom*[Title/Abstract] OR crico-thyroidotom*[Title/Abstract]))) OR ((thyrocricotom*[Title/Abstract] OR thyro-cricotom*[Title/Abstract]))) OR ((cricothyrotom*[Title/Abstract] OR crico-thyrotom*[Title/Abstract]))) OR coniotom*[Title/Abstract]) OR (("cricothyroid incision"[Title/Abstract] OR "cricothyroid cutting"[Title/Abstract] OR "cricothyroid puncture"[Title/Abstract] OR "cricothyroid dissection"[Title/Abstract] OR "crico-thyroid incision"[Title/Abstract] OR "crico-thyroid cutting"[Title/Abstract] OR "crico-thyroid puncture"[Title/Abstract] OR "crico-thyroid dissection"[Title/Abstract] OR "subthyroid incision"[Title/Abstract] OR "subthyroid cutting"[Title/Abstract] OR "subthyroid puncture"[Title/Abstract] OR "subthyroid dissection"[Title/Abstract] OR "sub-thyroid incision"[Title/Abstract] OR "sub-thyroid cutting"[Title/Abstract] OR "sub-thyroid puncture"[Title/Abstract] OR "sub-thyroid dissection"[Title/Abstract]))) OR "tracheostomy"[MeSH Terms]) OR "tracheotomy"[MeSH Terms]) OR ((tracheotom*[Title/Abstract] OR tracheostom*[Title/Abstract]))) OR ((minitracheostom*[Title/Abstract] OR mini-tracheostom*[Title/Abstract]))) OR ((minitracheotom*[Title/Abstract] OR mini-tracheotom*[Title/Abstract])))) AND ((Randomized Controlled Trial[ptyp] OR Observational Study[ptyp] OR Case Reports[ptyp] OR Comparative Study[ptyp] OR Multicenter Study[ptyp] OR systematic[sb] OR Review[ptyp]) AND adult[MeSH])))))) AND ((publisher[sb] OR pubmednotmedline[sb] OR inprocess[sb]))**

**CINAHL Search Strategy**

| **#** | **Query** | **Results** |
| --- | --- | --- |
| S54 | S49 AND S53 | 282 |
| S53 | S50 OR S51 OR S52 | 250,403 |
| S52 | TI ( (complication* or (adverse N2 (effect* or event* or condition*))) ) OR AB ( (complication* or (adverse N2 (effect* or event* or condition*))) ) | 211,165 |
| S51 | (MH "Adverse Health Care Event") | 5,994 |
| S50 | (MH "Postoperative Complications") | 55,154 |
| S49 | S23 AND S24 AND S48 | 964 |
| S48 | S39 OR S47 | 1,869,191 |
| S47 | S40 OR S41 OR S42 OR S43 OR S44 OR S45 OR S46 | 671,443 |
| S46 | TX observational N1 (study or studies) | 67,944 |
| S45 | TX (cohort N1 (study or studies)) | 120,269 |
| S44 | (MH "Cross Sectional Studies") | 151,489 |
| S43 | (MH "Nonconcurrent Prospective Studies") | 226 |
| S42 | (MH "Correlational Studies") | 22,342 |
| S41 | (MH "Case Control Studies+") | 69,555 |
| S40 | (MH "Prospective Studies") | 368,446 |
| S39 | S25 OR S26 OR S27 OR S28 OR S29 OR S30 OR S31 OR S32 OR S33 OR S34 OR S35 OR S36 OR S37 OR S38 | 1,394,751 |
| S38 | TX allocat* random* | 1,068 |
| S37 | MH "Quantitative Studies" | 21,278 |
| S36 | MH Placebos | 11,061 |
| S35 | TX placebo* | 105,377 |
| S34 | TX random* allocat* | 12,689 |
| S33 | MH "Random Assignment" | 51,958 |
| S32 | TX randomi* control* trial* | 215,283 |
| S31 | TX ( (singl* n1 blind*) or (singl* n1 mask*) ) | 19,281 |
| S30 | TX ( (doubl* n1 blind*) or (doubl* n1 mask*) ) | 1,001,203 |
| S29 | TX ( (tripl* n1 blind*) or (tripl* n1 mask*) ) | 609 |
| S28 | TX ( (trebl* n1 blind*) or (trebl* n1 mask*) ) | 12 |
| S27 | TX clinic* n1 trial* | 327,670 |
| S26 | PT Clinical trial | 86,770 |
| S25 | (MH "Clinical Trials+") | 252,079 |
| S24 | S14 OR S15 OR S16 OR S17 OR S18 OR S19 OR S20 OR S21 OR S22 | 6,021 |
| S23 | S1 OR S2 OR S3 OR S4 OR S5 OR S6 OR S7 OR S8 OR S9 OR S10 OR S11 OR S12 OR S13 | 521,975 |
| S22 | TI ( ((cricothyroid or crico-thyroid or subthyroid or sub-thyroid) N2 (incis* or cut* or punctur* or dissect*)) ) OR AB ( ((cricothyroid or crico-thyroid or subthyroid or sub-thyroid) N2 (incis* or cut* or punctur* or dissect*)) ) | 18 |
| S21 | TI ( tracheotom* or tracheostom* or minitracheostom* or mini-tracheostom* or minitracheotom* or mini-tracheotom* ) OR AB ( tracheotom* or tracheostom* or minitracheostom* or mini-tracheostom* or minitracheotom* or mini-tracheotom* ) | 4,141 |
| S20 | (MH "Tracheostomy Equipment and Supplies+") OR (MH "Tracheostomy and Ventilator Swallowing and Speaking Valve") | 602 |
| S19 | (MH "Tracheostomy Care") | 157 |
| S18 | (MH "Tracheostomy") | 3,629 |
| S17 | TI ( thyrocricotom* or thyro-cricotom* ) OR AB ( thyrocricotom* or thyro-cricotom* ) | 0 |
| S16 | TI coniotom* OR AB coniotom* | 2 |
| S15 | TI ( cricothyrotom* or crico-thyrotom* or cricothyroidotom* or crico-thyroidotom* ) OR AB ( cricothyrotom* or crico-thyrotom* or cricothyroidotom* or crico-thyroidotom* ) | 368 |
| S14 | (MH "Cricothyrotomy") | 142 |
| S13 | TI ( emergen* or "urgent care" or critical* or intensive* or trauma* or ambulatory or ICU* or CCU* or immediate* ) OR AB ( emergen* or "urgent care" or critical* or intensive* or trauma* or ambulatory or ICU* or CCU* or immediate* ) | 442,835 |
| S12 | (MH "Respiratory Care Units") | 175 |
| S11 | (MH "Intensive Care Units") | 31,522 |
| S10 | (MH "Ambulatory Care Nursing") OR (MH "Ambulatory Care Facilities") | 7,612 |
| S9 | (MH "Ambulatory Care") | 10,404 |
| S8 | (MH "Critical Care Nursing") OR (MH "Respiratory Nursing") | 19,617 |
| S7 | (MH "Emergency Care") | 22,120 |
| S6 | (MH "Critical Care") | 19,049 |
| S5 | (MH "Emergency Treatment") | 446 |
| S4 | (MH "Emergencies") | 8,642 |
| S3 | (MH "Trauma Nursing") OR (MH "Emergency Nursing") | 13,687 |
| S2 | (MH "Emergency Medical Services") OR (MH "Emergency Service") OR (MH "Trauma Centers") | 70,325 |
| S1 | (MH "Emergency Medicine") | 10,156 |
