# Supplementary material for: Complications of cricothyroidotomy versus tracheostomy in emergency surgical airway management: a systematic review
Source: BMC Anesthesiol. 2020 Aug 27;20:216. doi: 10.1186/s12871-020-01135-2 (PMC7450579; doi:10.1186/s12871-020-01135-2)
Supplement: Supplementary file 2 — Additional file 2. [file 12871_2020_1135_MOESM2_ESM.docx]

**APPENDIX 2**

**Minor complications (**evolving to spontaneous remission and/or not requiring intervention and/or not persisting chronically)

- transient hypotension (defined as systolic blood pressure below 90 mmHg for less than 5 minutes and no needs for vasopressors);
- transient hypertension (defined as systolic blood pressure above 160 mmHg for less than 5 minutes and no needs for vasopressors);
- transient acute hypoxemia (defined as oxygen peripheral saturation below 90% for less than 5 minutes as measured by the pulse oximeter)
- atelectasis
- inadvertent cuff puncture; cuff-related problems
- localized minor bleeding (self-limiting bleeding or bleeding successfully treated with local compression); instillation of topical vasoconstrictive agents; and/or electrocauterization
- localized subcutaneous emphysema without evidence of pneumothorax or pneumomediastinum
- local infections not causing sepsis, incision revision

**Major complications (**requiring intervention and/or persisting chronically)

- Laryngeal-Tracheal lesion, subglottic stenosis, tracheo-cutaneous fistula, tracheal granulation, scarring at operating site, false passage/incorrect cannulation, cannula/tube obstruction, esophageal injury, tracheoesophageal fistula, accidental decannulation, tracheomalacia, retropharyngeal abscess, fire in airway
- reintubation
- major bleeding requiring surgical exploration
- procedure-related deaths
- pneumothorax, pneumomediastinum, aspiration, pneumonia, pulmonary edema
- cardiac arrest, hypotension needing vasopressors, myocardium infarction
- acute hypoxemia (oxygen peripheral saturation below 90% for more than 5 minutes)
- major bleeding causing hypoxemia and/or requiring revision
- procedure-related sepsis
